# Serum magnesium and major adverse cardiovascular events in patients with diabetic foot ulcers: a retrospective cohort study

**DOI:** 10.3389/fendo.2026.1938560

**Published:** 2026-09-11

**Authors:** Lulu Liu, Yukun Tao, Jinzheng Hou, Guangxin Zhou, Li Xiao, Huijuan Zhu, Di Zhu, Da Zhang

**Affiliations:** Department of Endocrinology, Air Force Medical Center, Air Force Medical University, Beijing, China

**Keywords:** cohort study, diabetic foot ulcer, magnesium, major adverse cardiovascular events, nonlinear relationship, retrospective study

## Abstract

**Background and aims:**

Patients with diabetic foot ulcers (DFU) have a high cardiovascular risk. We evaluated the association of baseline total serum magnesium (Mg) with major adverse cardiovascular events (MACE) without presuming a causal or therapeutic effect.

**Methods:**

This single-center retrospective cohort included 1,176 patients with active DFU admitted from 2015 through 2022. The endpoint was the first MACE, defined as cardiovascular death, nonfatal myocardial infarction, or nonfatal stroke. Cox models estimated hazard ratios (HRs) per 0.1 mmol/L and per standard deviation (SD) of Mg and by data-defined quartile groups. The extended model additionally adjusted for HbA1c, eGFR, albumin, log-transformed C-reactive protein, HDL-C, and LDL-C. Restricted cubic splines used four knots. Competing-risk, renal-function, complete-case, prior-cardiovascular-disease, time-specific, and propensity-score-matched analyses were performed.

**Results:**

During a median follow-up of 3.6 years, 304 MACE events occurred, including 216 cardiovascular deaths and 88 combined nonfatal myocardial infarctions or strokes. In the extended model, each 0.1 mmol/L higher Mg was associated with lower MACE hazard (HR 0.87, 95% CI 0.80–0.95; p=0.002); the HR per SD was 0.83 (95% CI 0.74–0.94; p=0.002). The spline model supported an overall (p<0.001) and nonlinear (p<0.001) association. An exploratory two-change-point model selected 0.83 and 0.99 mmol/L, but profile-likelihood regions were broad (0.75–0.85 and 0.95–1.10 mmol/L). Results were directionally consistent in competing-risk and other sensitivity analyses. In 324 matched pairs, the high (>0.80 mmol/L) versus low (≤0.80 mmol/L) contrast was associated with lower MACE hazard (pair-stratified HR 0.60, 95% CI 0.39–0.92; p=0.020), whereas the continuous estimate was less precise (HR 0.85, 95% CI 0.71–1.01; p=0.070).

**Conclusions:**

Baseline serum Mg was independently associated with MACE risk in patients with DFU, with an exploratory nonlinear pattern. The data-derived change points are not therapeutic boundaries and require external prospective validation. These observational findings do not establish benefit from Mg supplementation.

## 1 Introduction

Diabetic foot ulcer (DFU) is a severe chronic complication of diabetes and is associated with amputation, impaired quality of life, and high mortality (1). Patients with DFU also have a substantial burden of cardiovascular morbidity and mortality (2). Major adverse cardiovascular events (MACE)—commonly cardiovascular death, nonfatal myocardial infarction, and nonfatal stroke—are therefore clinically important outcomes in this population (3, 4). Identification of reproducible cardiovascular risk markers could improve risk stratification, although observational associations alone cannot define treatment targets.

Magnesium is an abundant intracellular cation involved in vascular tone, endothelial function, myocardial excitability, glucose metabolism, and inflammatory pathways (5–9). Population studies and studies in high-risk cardiovascular cohorts have generally reported inverse associations between circulating Mg and cardiovascular outcomes (10–13). Evidence that perioperative Mg can influence selected arrhythmic outcomes does not establish that supplementation prevents MACE in patients with DFU (14–17).

The association between serum Mg and MACE has not been adequately characterized in patients with active DFU. We therefore examined the continuous, categorical, and potentially nonlinear association between a single baseline total serum Mg measurement and subsequent MACE. We also used clinically informed adjustment and performed complementary analyses addressing renal function, competing mortality, missing data, previous cardiovascular disease, possible nonproportional hazards, and propensity-score balance. Any change points were treated as exploratory and data-derived.

## 2 Materials and methods

### 2.1 Study design and participants

**Figure 1:**
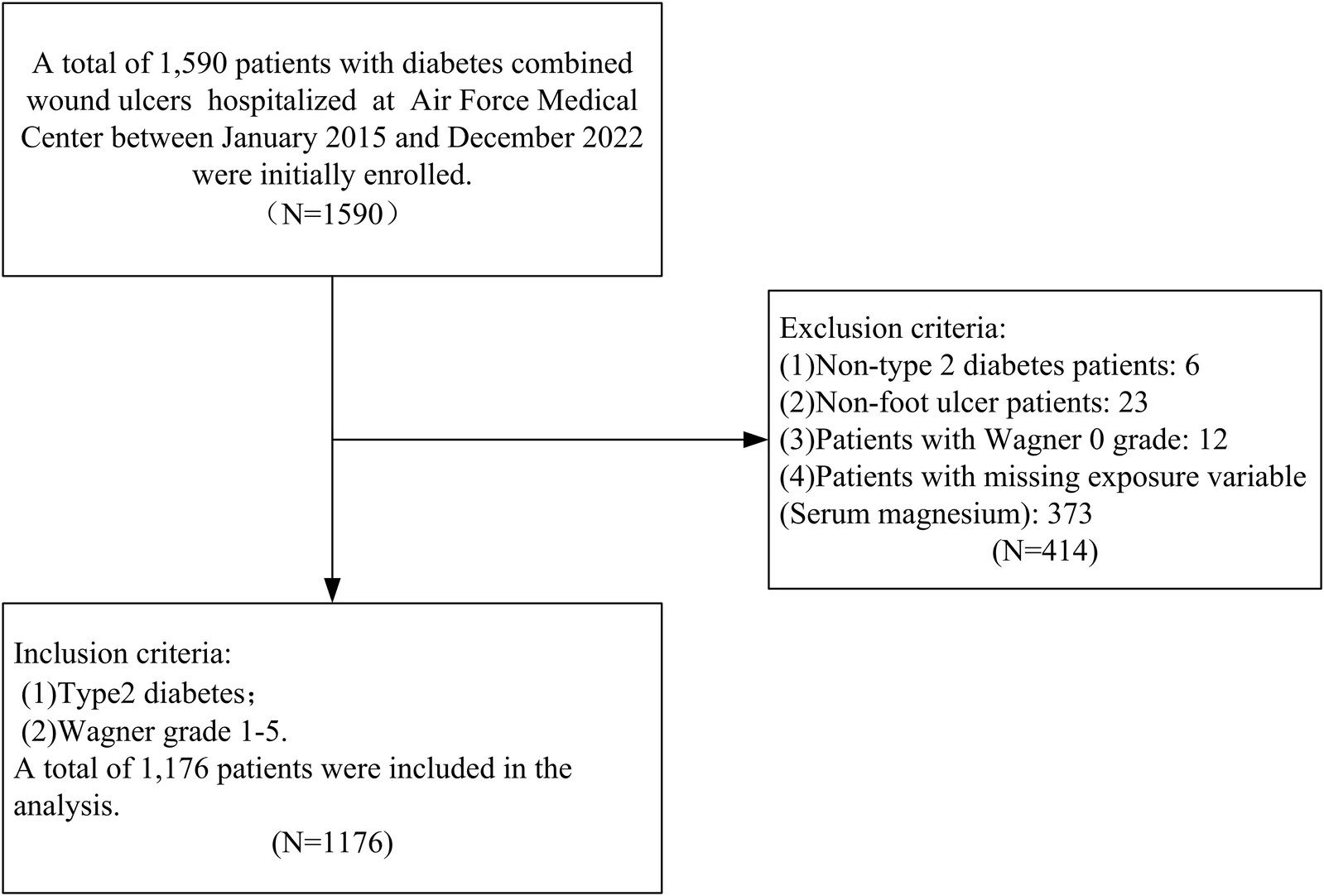
Participant selection and analysis cohorts. Of 1,590 screened patients, 414 were excluded and 1,176 were included in the primary imputed cohort. The figure also identifies the complete-case cohort and the revised propensity-score-matched sensitivity cohort. DFU, diabetic foot ulcer; MACE, major adverse cardiovascular events; PSM, propensity-score matching; RCS, restricted cubic spline.

This single-center retrospective cohort used electronic medical records for 1,590 patients treated for diabetic foot disease at the Air Force Medical Center between January 2015 and December 2022. For patients with more than one hospitalization, the first eligible admission was the index hospitalization. All included participants had type 2 diabetes and an active foot ulcer of Wagner grade 1–5. Exclusions were non-type 2 diabetes (n=6), no active ulcer (n=23), Wagner grade 0 (n=12), and absence of a baseline serum Mg result (n=373), leaving 1,176 patients (**Figure 1**). The supplied analysis files did not contain baseline variables for the 373 patients excluded for missing Mg; an included-versus-excluded comparison could therefore not be performed.

The study followed the Declaration of Helsinki and was approved by the Ethics Committee of the Air Force Medical Center (AFMC-2022-232-PJ01). The committee waived individual informed consent for this retrospective analysis.

### 2.2 Serum magnesium measurement

The exposure was total serum Mg measured with a xylidyl blue assay on a Beckman Coulter AU5800 automated biochemical analyzer. The value was obtained from the first fasting blood sample during the index hospitalization and was analyzed continuously and categorically. The analysis dataset did not retain the exact interval from admission or initiation of intravenous fluids, insulin, antibiotics, or other treatments, and it did not include the institution-specific laboratory reference interval. These limitations were considered when interpreting a single baseline measurement.

### 2.3 Endpoint and follow-up

The primary endpoint was the first MACE, defined as cardiovascular death, nonfatal myocardial infarction (MI), or nonfatal stroke, consistent with standard cardiovascular endpoint concepts (4). Follow-up sources in the original study included structured telephone or short-message follow-up, clinical records, and the Centers for Disease Control death registry. Follow-up was administratively censored on 31 March 2024. Time at risk extended from the index admission to first MACE or censoring.

The archived analysis dataset retained a composite MACE indicator and cardiovascular-death indicator; nonfatal MI and stroke were not separately encoded. Therefore, component reporting is limited to cardiovascular death and combined nonfatal MI/stroke. For data-quality reconciliation, cardiovascular-death coding took precedence over a discordant generic all-cause-death field. Noncardiovascular death was then defined as death without cardiovascular death or MACE and was used as the competing event. The dataset did not retain the event-level diagnostic subcriteria, formal adjudication status, source-discrepancy documentation, or a separate loss-to-follow-up indicator.

### 2.4 Covariates

Baseline covariates were age, sex, body mass index (BMI), smoking, duration of diabetes, ulcer duration, diabetic peripheral neuropathy (DPN), diabetic retinopathy (DR), diabetic nephropathy (DN), peripheral artery disease (PAD), hyperlipidemia, hypertension, coronary heart disease (CHD), previous stroke, Wagner grade, glycated hemoglobin A1c (HbA1c), fasting plasma glucose (FPG), serum creatinine, estimated glomerular filtration rate (eGFR), serum uric acid, total cholesterol, triglycerides, high-density lipoprotein cholesterol (HDL-C), low-density lipoprotein cholesterol (LDL-C), albumin, C-reactive protein (CRP), superoxide dismutase (SOD), and triglyceride-glucose (TyG) index. BMI was calculated as kg/m². Laboratory variables came from the first fasting sample during the index hospitalization.

### 2.5 Missing data

Missingness in measured covariates ranged from 0.2% for albumin to 25.3% for DN (**Supplementary Table 1**). MissForest was used once to create a single completed dataset; it was not a formal multiple-imputation procedure and no Rubin-rule pooling was performed (18). Serum Mg, MACE status, and follow-up time were not imputed. The imputed cohort was used for the primary analysis, and a complete-case analysis was performed as a sensitivity analysis.

### 2.6 Statistical analysis

Because ties occurred at empirical percentiles, the categorical groups were defined by observed Mg values as Q1 <0.80 mmol/L, Q2 0.80–0.89 mmol/L, Q3 0.90–0.99 mmol/L, and Q4 ≥1.00 mmol/L. Continuous variables were summarized as mean ± SD when approximately symmetric and as median (interquartile range) when skewed; categorical variables were summarized as n (%). Across-group comparisons used one-way analysis of variance, Kruskal–Wallis tests, or chi-square tests, as appropriate. Kaplan–Meier curves described MACE-free survival, with groups compared by the log-rank test.

Cox proportional hazards models used Efron handling of tied event times. Mg was modeled per 0.1 mmol/L, per one SD (0.133 mmol/L), and by category with Q1 as reference. The crude model was unadjusted; Model 1 adjusted for age and sex; Model 2 adjusted for age, sex, BMI, smoking, duration of diabetes, ulcer duration, DPN, PAD, DR, DN, hypertension, CHD, previous stroke, hyperlipidemia, and Wagner grade; and Model 3 additionally adjusted for HbA1c, eGFR, albumin, log(CRP + 1), HDL-C, and LDL-C. Covariates for the revised analysis were selected on clinical grounds for relevance to Mg status and cardiovascular prognosis rather than by outcome-based screening. To limit collinearity and overadjustment, Model 3 used eGFR rather than simultaneously adding serum creatinine, HbA1c rather than FPG, and HDL-C/LDL-C rather than all correlated lipid and TyG measures. Model 3 contained 24 regression degrees of freedom including Mg, corresponding to approximately 12.7 MACE events per parameter.

Nonlinearity was assessed in Model 3 with a restricted cubic spline using knots at the 5th, 35th, 65th, and 95th percentiles (0.70, 0.86, 0.96, and 1.10 mmol/L) and 0.90 mmol/L as the reference. Likelihood-ratio tests compared the full spline with the model without Mg (overall association) and with a linear-Mg model (nonlinearity). The curve was displayed from the 1st to 99th percentiles. As an exploratory analysis, a formal continuous two-change-point Cox model systematically searched candidate values across the central observed Mg distribution, requiring at least 50 participants and 15 events per segment. The best log-likelihood model was compared with a linear model; approximate 95% profile-likelihood regions described threshold uncertainty. Because change points were selected in the same dataset, the likelihood-ratio p-value is descriptive and no internal validation was claimed.

The proportional hazards (PH) assumption was evaluated for Mg and principal covariates using scaled Schoenfeld-residual score tests. Because Mg and the global model showed evidence of nonproportionality, time-specific Cox models were estimated for 0–2 years and >2 years. Noncardiovascular death was addressed in a Fine–Gray subdistribution-hazard sensitivity analysis.

Prespecified exploratory subgroup analyses evaluated sex, age, BMI, PAD, CHD, hypertension, DPN, DN, DR, previous stroke, and Wagner grade. Interaction p-values were derived from likelihood-ratio comparisons of models with and without Mg-by-subgroup interaction terms. Subgroup-specific statistical significance was not interpreted as evidence of between-group heterogeneity without a corresponding interaction.

Propensity-score matching (PSM) was retained only as a sensitivity analysis. The 0.80 mmol/L cutoff (low ≤0.80; high >0.80 mmol/L) had originally been selected using MACE in X-tile and was therefore explicitly considered outcome-driven. Propensity scores were estimated from demographic, clinical, Wagner-grade, glycemic, renal, inflammatory, nutritional, lipid, SOD, and TyG covariates. Optimal 1:1 matching without replacement used a caliper of 0.20 SD of the propensity-score logit. Balance was assessed with absolute standardized mean differences (SMDs), with <0.10 as the target. Outcome models were stratified by matched pair. Two-sided p<0.05 indicated statistical significance; exact p-values are reported to three decimals unless p<0.001.

## 3 Results

### 3.1 Baseline characteristics and magnesium distribution

The cohort included 1,176 patients: Q1 n=183, Q2 n=286, Q3 n=324, and Q4 n=383. Mean age was 61.96 ± 11.59 years, 72.3% were male, and 53.8% had Wagner grade 4–5 ulcers (**Table 1**). Age, BMI, smoking, durations, major diabetes complications, and cardiovascular comorbidities did not differ materially across Mg groups. Higher-Mg groups had lower HbA1c, FPG, eGFR, and CRP and higher serum creatinine, HDL-C, albumin, and SOD, among other differences, supporting extended confounder adjustment. Serum Mg had a mean of 0.908 ± 0.133 mmol/L, median 0.90 mmol/L (IQR 0.80–1.00), and range 0.20–1.50 mmol/L; the 5th, 95th, and 99th percentiles were 0.70, 1.10, and 1.20 mmol/L, respectively.

**Table 1:** Baseline characteristics by serum magnesium category Q1 <0.80 mmol/L; Q2 0.80–0.89 mmol/L; Q3 0.90–0.99 mmol/L; Q4 ≥1.00 mmol/L.

Characteristic	Overall (n=1,176)	Q1 (n=183)	Q2 (n=286)	Q3 (n=324)	Q4 (n=383)	p
Age, years	61.96 ± 11.59	61.35 ± 11.74	61.76 ± 11.72	62.66 ± 10.71	61.81 ± 12.15	0.607
Male sex	850 (72.28)	119 (65.03)	209 (73.08)	233 (71.91)	289 (75.46)	0.077
BMI, kg/m²	24.56 ± 3.31	24.46 ± 3.67	24.37 ± 3.29	24.68 ± 3.45	24.65 ± 3.02	0.603
Current/former smoking	488 (41.50)	69 (37.70)	124 (43.36)	131 (40.43)	164 (42.82)	0.589
Duration of diabetes, years	15.00 (9.00, 20.00)	15.00 (10.00, 20.00)	15.00 (10.00, 20.00)	15.00 (8.75, 20.00)	15.00 (7.00, 20.00)	0.364
Ulcer duration, months	30.00 (20.00, 100.00)	30.00 (20.00, 100.00)	30.00 (20.00, 90.00)	30.00 (20.00, 100.00)	30.00 (20.00, 100.00)	0.893
Diabetic peripheral neuropathy	1040 (88.44)	158 (86.34)	249 (87.06)	290 (89.51)	343 (89.56)	0.542
Diabetic retinopathy	612 (52.04)	97 (53.01)	141 (49.30)	170 (52.47)	204 (53.26)	0.757
Diabetic nephropathy	676 (57.48)	110 (60.11)	166 (58.04)	186 (57.41)	214 (55.87)	0.811
Peripheral artery disease	838 (71.26)	122 (66.67)	201 (70.28)	234 (72.22)	281 (73.37)	0.392
Hyperlipidemia	830 (70.58)	133 (72.68)	217 (75.87)	220 (67.90)	260 (67.89)	0.082
Hypertension	818 (69.56)	130 (71.04)	211 (73.78)	221 (68.21)	256 (66.84)	0.240
Coronary heart disease	326 (27.72)	58 (31.69)	75 (26.22)	88 (27.16)	105 (27.42)	0.608
Previous stroke	247 (21.00)	36 (19.67)	45 (15.73)	76 (23.46)	90 (23.50)	0.055
HbA1c, %	8.81 (7.69, 10.20)	9.39 (7.80, 10.85)	9.12 (7.90, 10.47)	8.74 (7.70, 9.91)	8.60 (7.40, 9.80)	<0.001
FPG, mmol/L	7.90 (6.00, 10.50)	8.10 (6.38, 10.78)	8.09 (6.20, 11.02)	8.00 (6.20, 10.70)	7.50 (5.80, 10.10)	0.047
Serum creatinine, µmol/L	70.00 (56.00, 93.00)	67.00 (54.50, 85.00)	67.00 (53.00, 89.75)	69.00 (55.75, 88.00)	76.08 (58.00, 104.50)	<0.001
eGFR, mL/min/1.73 m²	92.48 (71.23, 104.96)	92.89 (76.54, 106.56)	94.55 (75.59, 107.57)	92.69 (75.09, 105.04)	88.59 (60.96, 102.80)	<0.001
Serum uric acid, µmol/L	296.00 (234.00, 369.25)	304.00 (236.00, 372.00)	280.00 (217.25, 353.75)	289.00 (242.75, 361.00)	309.00 (240.50, 381.50)	0.013
Total cholesterol, mmol/L	3.80 (3.11, 4.52)	3.53 (2.96, 4.11)	3.84 (3.02, 4.58)	3.79 (3.13, 4.56)	3.96 (3.26, 4.60)	<0.001
Triglycerides, mmol/L	1.26 (0.97, 1.73)	1.21 (0.94, 1.59)	1.25 (1.00, 1.70)	1.27 (0.98, 1.86)	1.27 (0.95, 1.79)	0.351
HDL-C, mmol/L	0.91 (0.75, 1.09)	0.82 (0.68, 1.02)	0.87 (0.74, 1.07)	0.92 (0.76, 1.10)	0.95 (0.79, 1.13)	<0.001
LDL-C, mmol/L	2.20 (1.67, 2.77)	1.99 (1.58, 2.45)	2.19 (1.59, 2.78)	2.25 (1.69, 2.83)	2.26 (1.73, 2.79)	0.004
Albumin, g/L	37.39 (33.40, 40.40)	36.10 (31.47, 39.15)	36.10 (32.50, 39.68)	37.60 (33.58, 40.50)	38.40 (35.15, 41.60)	<0.001
CRP, mg/L	12.50 (3.80, 49.78)	25.80 (5.83, 65.88)	15.83 (4.01, 63.63)	12.10 (3.90, 46.08)	7.91 (3.11, 29.45)	<0.001
SOD, U/mL	112.55 ± 27.78	108.56 ± 29.36	109.94 ± 27.73	112.49 ± 26.97	116.45 ± 27.32	0.003
TyG index	9.03 ± 0.66	9.01 ± 0.60	9.06 ± 0.68	9.06 ± 0.61	9.01 ± 0.72	0.642
Wagner grade 1	48 (4.08)	6 (3.28)	14 (4.90)	14 (4.32)	14 (3.66)	0.067
Wagner grade 2	171 (14.54)	26 (14.21)	27 (9.44)	45 (13.89)	73 (19.06)	
Wagner grade 3	324 (27.55)	47 (25.68)	79 (27.62)	95 (29.32)	103 (26.89)	
Wagner grade 4-5	633 (53.83)	104 (56.83)	166 (58.04)	170 (52.47)	193 (50.39)	

Values are mean ± SD, median (IQR), or n (%), consistently for each variable. P values compare all four groups by one-way ANOVA, Kruskal–Wallis test, or chi-square test. Wagner grades 4 and 5 were combined because grade 5 was sparse. Values shown are from the completed (single MissForest-imputed) analysis dataset. BMI, body mass index; CHD, coronary heart disease; CRP, C-reactive protein; DPN, diabetic peripheral neuropathy; DR, diabetic retinopathy; DN, diabetic nephropathy; eGFR, estimated glomerular filtration rate; FPG, fasting plasma glucose; HDL-C, high-density lipoprotein cholesterol; IQR, interquartile range; LDL-C, low-density lipoprotein cholesterol; PAD, peripheral artery disease; SOD, superoxide dismutase; TyG, triglyceride-glucose index.

### 3.2 Event counts and unadjusted survival

**Figure 2:**
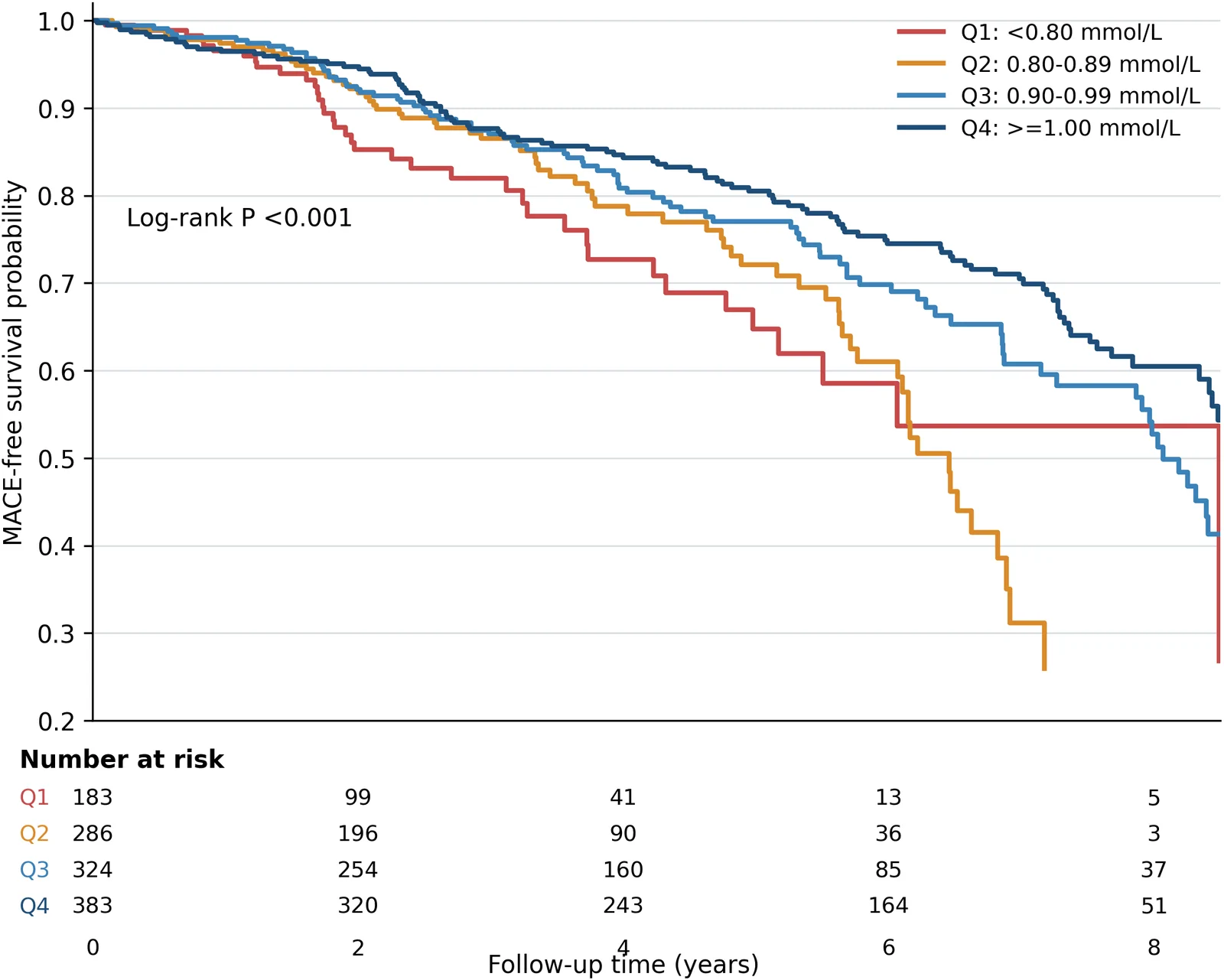
Kaplan–Meier curves for MACE-free survival by baseline serum magnesium category: Q1 <0.80 mmol/L, Q2 0.80–0.89 mmol/L, Q3 0.90–0.99 mmol/L, and Q4 ≥1.00 mmol/L. The time axis is in years and the risk table is shown below the curves. Log-rank p<0.001. The sparse late risk sets should be interpreted cautiously.

Over a median 1,297 days (3.55 years) of follow-up, 304 patients experienced MACE (25.9%): 216 cardiovascular deaths and 88 combined nonfatal MI/stroke events. There were 153 noncardiovascular deaths. MACE counts across Q1–Q4 were 38, 69, 92, and 105, respectively; corresponding cardiovascular-death counts were 24, 44, 64, and 84 (**Table 2**). Kaplan–Meier curves differed across groups (log-rank p<0.001; **Figure 2**).

**Table 2:** Outcome counts and follow-up by serum magnesium category.

Group	n	MACE	Cardiovascular death	Nonfatal MI or stroke, combined	Non-cardiovascular death	Median follow-up, days
Overall	1,176	304	216	88	153	1,297
Q1	183	38	24	14	19	755
Q2	286	69	44	25	35	1,058
Q3	324	92	64	28	36	1,448
Q4	383	105	84	21	63	1,906

The archived dataset did not encode nonfatal MI and nonfatal stroke separately. Noncardiovascular death was defined after cardiovascular-death/all-cause-death reconciliation and was used as the competing event in the Fine–Gray analysis. A separate loss-to-follow-up indicator was not available.

### 3.3 Multivariable association

In Model 3, each 0.1 mmol/L higher Mg was independently associated with lower MACE hazard (HR 0.87, 95% CI 0.80–0.95; p=0.002), and the HR per SD was 0.83 (95% CI 0.74–0.94; p=0.002) (**Table 3**). Relative to Q1, Model 3 HRs were 0.98 (95% CI 0.66–1.48; p=0.940) for Q2, 0.70 (95% CI 0.47–1.04; p=0.079) for Q3, and 0.47 (95% CI 0.31–0.71; p<0.001) for Q4. The per-0.1 mmol/L HR moved from 0.84 in the age/sex model to 0.87 after clinical adjustment, consistent with attenuation toward the null after accounting for covariates correlated with Mg and MACE; the extended biochemical adjustment changed the estimate minimally.

**Table 3:** Association of baseline serum magnesium with MACE.

Exposure	Crude HR (95% CI)	p	Model 1 HR (95% CI)	p	Model 2 HR (95% CI)	p	Model 3 HR (95% CI)	p
Per 0.1 mmol/L	0.86 (0.79–0.94)	<0.001	0.84 (0.77–0.91)	<0.001	0.87 (0.80–0.95)	0.001	0.87 (0.80–0.95)	0.002
Per 1 SD (0.133 mmol/L)	0.82 (0.73–0.92)	<0.001	0.79 (0.71–0.89)	<0.001	0.83 (0.74–0.93)	0.001	0.83 (0.74–0.94)	0.002
Q2 vs Q1	0.94 (0.63–1.39)	0.748	0.93 (0.63–1.38)	0.720	0.96 (0.64–1.44)	0.855	0.98 (0.66–1.48)	0.940
Q3 vs Q1	0.66 (0.45–0.97)	0.036	0.63 (0.43–0.93)	0.020	0.68 (0.46–1.01)	0.056	0.70 (0.47–1.04)	0.079
Q4 vs Q1	0.49 (0.34–0.72)	<0.001	0.44 (0.30–0.64)	<0.001	0.49 (0.33–0.72)	<0.001	0.47 (0.31–0.71)	<0.001

All models included n=1,176 and 304 MACE events. Crude: unadjusted. Model 1: age and sex. Model 2: age, sex, BMI, smoking, duration of diabetes, ulcer duration, DPN, PAD, DR, DN, hypertension, CHD, previous stroke, hyperlipidemia, and Wagner grade. Model 3: Model 2 plus HbA1c, eGFR, albumin, log(CRP+1), HDL-C, and LDL-C. Q1 is the reference category. HRs are average effects over follow-up; the PH sensitivity analyses are reported in **Supplementary Tables 3**, **4**.

### 3.4 Exploratory nonlinear and change-point analyses

**Figure 3:**
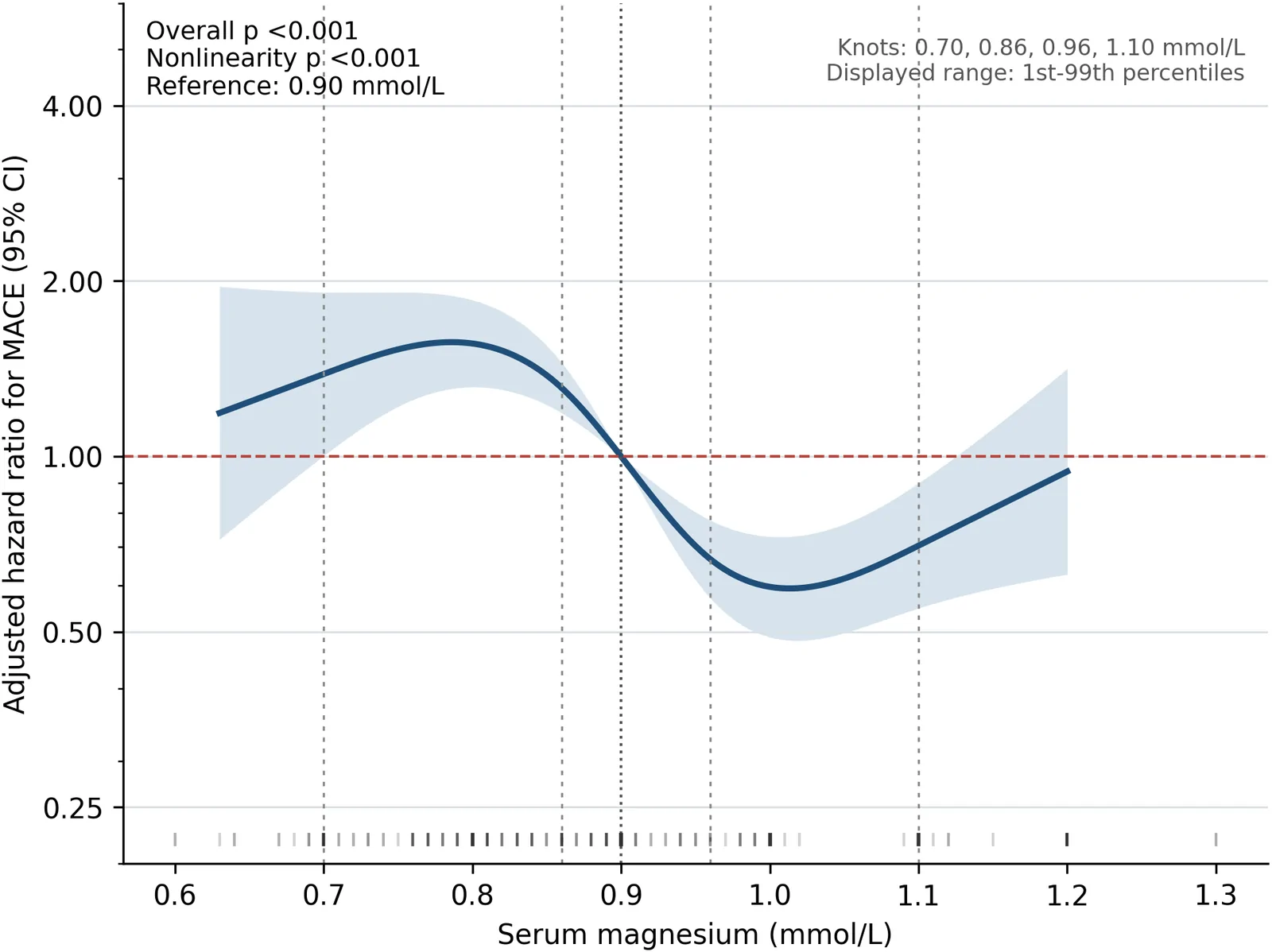
Restricted cubic spline for baseline serum magnesium and MACE. The curve is adjusted as Model 3 for age, sex, BMI, smoking, duration of diabetes, ulcer duration, DPN, PAD, DR, DN, hypertension, CHD, previous stroke, hyperlipidemia, Wagner grade, HbA1c, eGFR, albumin, log(CRP + 1), HDL-C, and LDL-C. Knots were placed at 0.70, 0.86, 0.96, and 1.10 mmol/L (5th, 35th, 65th, and 95th percentiles); 0.90 mmol/L was the reference. The curve is displayed from the 1st to 99th percentiles. Overall p<0.001; nonlinear p<0.001. Shading denotes the 95% CI.

The Model 3 spline supported both an overall association (p<0.001) and nonlinearity (p<0.001) (**Figure 3**). The fitted curve reached its lowest point near 1.00 mmol/L, with wide uncertainty at the distributional extremes. The two-change-point model selected 0.83 and 0.99 mmol/L; approximate profile-likelihood regions were 0.75–0.85 and 0.95–1.10 mmol/L. HRs per 0.1 mmol/L were 1.17 (95% CI 0.90–1.51; p=0.245) below 0.83, 0.51 (95% CI 0.40–0.66; p<0.001) from 0.83 to <0.99, and 1.28 (95% CI 1.03–1.60; p=0.024) at ≥0.99 mmol/L. These estimates arose from the same data used to select the change points and are exploratory. The upper tail was limited: 39 participants (19 MACE) had Mg ≥1.20 mmol/L and only 5 (4 MACE) had Mg ≥1.30 mmol/L (**Supplementary Tables 5**, **6**).

### 3.5 Model assumptions and sensitivity analyses

Schoenfeld-residual tests suggested nonproportionality for Mg (p=0.024), eGFR (p=0.005), log-CRP (p=0.017), DPN (p<0.001), and the global model (p=0.005) (**Supplementary Table 4**). The Model 3 HR for Mg was 0.93 (95% CI 0.80–1.08; p=0.335) during 0–2 years and 0.84 (95% CI 0.75–0.93; p=0.001) after 2 years; the primary Cox estimate should therefore be interpreted as an average association over follow-up. In the Fine–Gray analysis treating noncardiovascular death as competing, the subdistribution HR per 0.1 mmol/L was 0.91 (95% CI 0.83–0.99; p=0.034).

The association remained in the same direction in the complete-case analysis (n=598; 141 events; HR 0.85, 95% CI 0.75–0.97; p=0.015), after excluding eGFR <30 mL/min/1.73 m² (n=1,101; 272 events; HR 0.80, 95% CI 0.72–0.89; p<0.001), among patients without previous CHD or stroke (n=710; 152 events; HR 0.82, 95% CI 0.71–0.95; p=0.008), and among those with previous CHD or stroke (n=466; 152 events; HR 0.90, 95% CI 0.81–1.01; p=0.075). Renal-stratum estimates were directionally similar but imprecise for eGFR <60 mL/min/1.73 m² (**Supplementary Table 3**). Dialysis status was not available.

After PSM retained 324 pairs (n=648; 167 MACE), discarding 10 low-Mg and 518 high-Mg patients. All post-match absolute SMDs were <0.10 (maximum 0.100; **Supplementary Table 2** and **Supplementary Figure 1**). In Cox analysis stratified by matched pair, high (>0.80 mmol/L) versus low (≤0.80 mmol/L) Mg was associated with lower MACE hazard (HR 0.60, 95% CI 0.39–0.92; p=0.020). The continuous per-0.1 mmol/L estimate was directionally similar but did not reach conventional significance (HR 0.85, 95% CI 0.71–1.01; p=0.070).

### 3.6 Exploratory subgroup analyses

**Figure 4:**
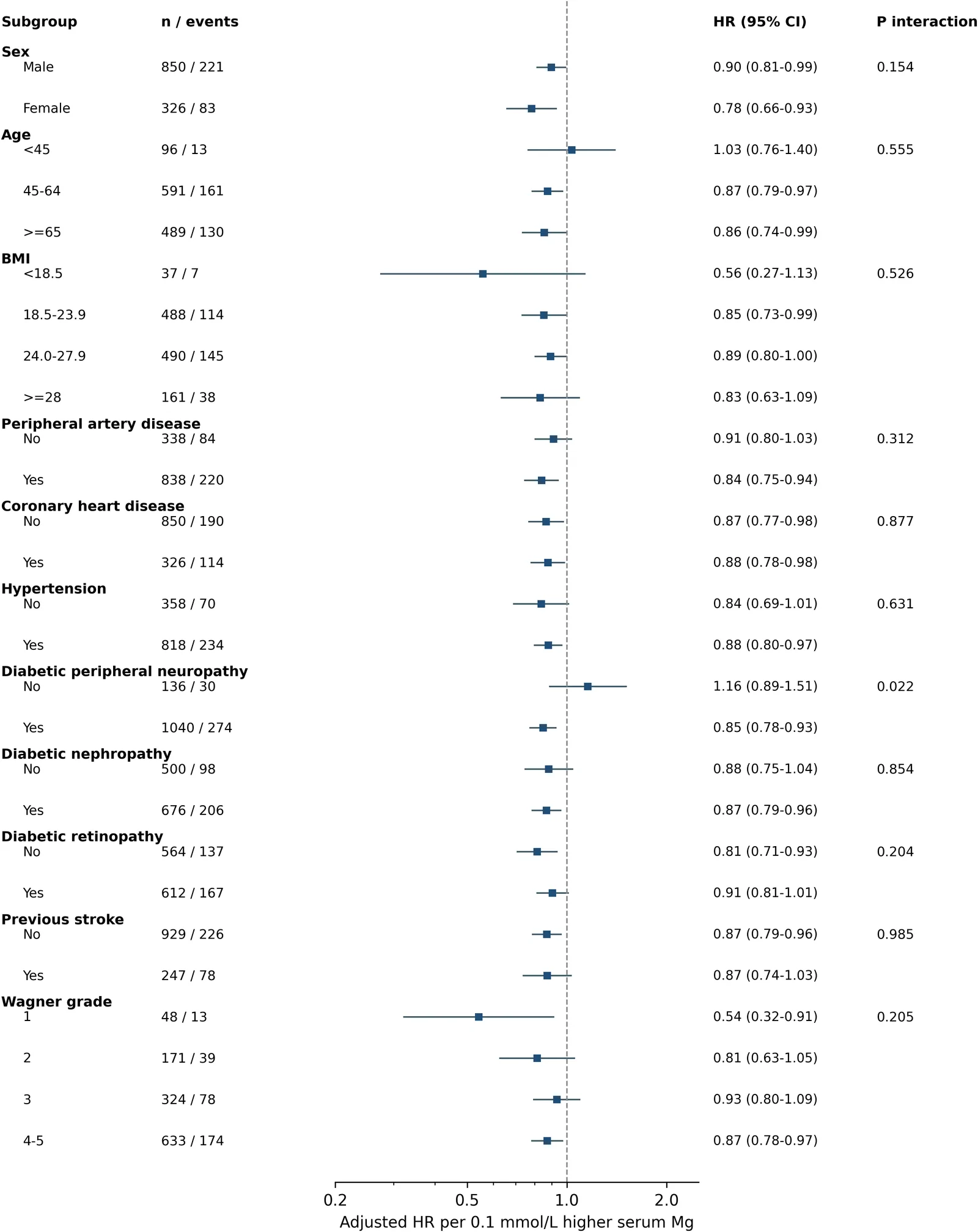
Exploratory subgroup associations per 0.1 mmol/L higher baseline serum magnesium. Points and horizontal bars are adjusted HRs and 95% CIs; interaction p-values test Mg-by-subgroup heterogeneity. The DPN interaction (p=0.022) should be interpreted cautiously because the no-DPN subgroup was small and imprecise. DPN, diabetic peripheral neuropathy; HR, hazard ratio; MACE, major adverse cardiovascular events.

Interaction tests were nonsignificant for sex, age, BMI, PAD, CHD, hypertension, DN, DR, previous stroke, and Wagner grade (all p for interaction>0.05; **Figure 4**). The DPN interaction was nominally significant (p=0.022): the HR was 1.16 (95% CI 0.89–1.51) among 136 patients without DPN and 0.85 (95% CI 0.78–0.93) among 1,040 with DPN.

## 4 Discussion

In this retrospective cohort of 1,176 patients with active DFU, 304 MACE events occurred during a median follow-up of 3.55 years. After adjustment for demographic, clinical, and selected biochemical confounders, each 0.1 mmol/L higher baseline total serum Mg was associated with a 13% lower average hazard of MACE (HR 0.87, 95% CI 0.80–0.95), and the corresponding HR per SD was 0.83 (95% CI 0.74–0.94). Compared with Q1, Q4 was associated with a lower hazard (HR 0.47, 95% CI 0.31–0.71). The direction of association was maintained in competing-risk, complete-case, and renal-function analyses and in patients with and without previous cardiovascular disease. The pair-stratified PSM analysis was directionally concordant, although its continuous estimate was less precise. Because the PH assumption was not fully satisfied, the principal Cox estimate should be interpreted as an average association over follow-up. The spline analysis supported an overall and nonlinear association, with a fitted risk nadir near 1.00 mmol/L and exploratory changes in slope near 0.83 and 0.99 mmol/L. However, the broad profile-likelihood regions, sparse upper tail, and same-sample selection of these change points preclude interpreting them as precise clinical or therapeutic boundaries.

These findings are broadly consistent with evidence from other populations. In participants with atrial fibrillation in the ARIC cohort, Li et al. reported generally lower rates of several cardiovascular endpoints at higher circulating Mg concentrations, although estimates were imprecise and continuous associations were not uniformly evident (5). Other observational studies have linked low circulating Mg to ischemic heart disease, cardiovascular and all-cause mortality, and MACE after acute myocardial infarction (11–13). Additional ARIC analyses associated low serum Mg with coronary disease, incident heart failure, and sudden cardiac death (19–21). The present study extends these observations to patients with active DFU, a population with a substantial overall and cardiovascular mortality burden (1, 2), and evaluates clinically scaled continuous estimates, categorical contrasts, and the dose-response shape while accounting for competing mortality, renal function, missing data, previous cardiovascular disease, and propensity-score balance. The attenuation from Model 1 to Model 2 indicates that part of the age- and sex-adjusted association was explained by measured clinical covariates. The near-identical Model 2 and Model 3 estimates suggest that the association was not further explained to a large extent by the selected renal, glycemic, inflammatory, nutritional, and lipid variables. Nevertheless, this pattern does not exclude residual or unmeasured confounding, and the exploratory nonlinear shape should be viewed as an extension of prior observational evidence rather than proof of an optimal magnesium range.

Several biologically plausible pathways may underlie—or confound—the observed association. Magnesium contributes to vascular smooth-muscle tone, endothelial signaling, myocardial ion transport and electrical stability, insulin action, inflammatory regulation, and oxidative balance (22, 23). Low magnesium status has also been associated with common microvascular and macrovascular complications of diabetes. (24) These processes are especially relevant in DFU, in which peripheral arterial disease, infection, chronic inflammation, malnutrition, renal dysfunction, and advanced diabetes frequently coexist. In the present cohort, higher Mg groups had lower HbA1c, an established glycemic marker (25), and CRP and higher albumin and SOD, a pattern compatible with differences in metabolic control, inflammation, nutrition, and oxidative stress. Conversely, higher Mg also coincided with lower eGFR and higher serum creatinine, underscoring the potential influence of renal clearance on circulating Mg. Thus, baseline total serum Mg may be biologically relevant while simultaneously serving as a marker of broader cardio-renal and systemic illness (26). These baseline patterns cannot establish mediation, resolve confounding, or demonstrate that modifying Mg will improve cardiovascular outcomes.

From a clinical perspective, total serum Mg is inexpensive and routinely available and may warrant evaluation as one component of multivariable cardiovascular risk assessment in patients with DFU. For now, an abnormal value should prompt integrated evaluation of renal function, glycemic control, inflammation, nutritional status, and medications; serum Mg should not be interpreted as a stand-alone treatment target. Neither the exploratory change points near 0.83 and 0.99 mmol/L nor the outcome-derived 0.80 mmol/L cutoff used in the PSM analysis should guide magnesium supplementation or define a therapeutic range. Before clinical implementation, the association and dose-response shape should be replicated in external multicenter prospective cohorts with standardized sampling, serial total and, where feasible, ionized Mg measurements, detailed medication and DFU severity data, and adjudicated cardiovascular endpoints. Interventional trials would then be required to determine whether correcting magnesium deficiency or otherwise modifying Mg status changes MACE risk.

## 5 Limitations

This study has several limitations. First, its single-center retrospective design precludes causal inference and permits residual confounding. Medication exposures that can influence Mg or cardiovascular risk—including proton-pump inhibitors, loop or thiazide diuretics, Mg supplements, statins, antiplatelet agents, and glucose-lowering drugs—were not available in the supplied dataset. Dialysis status and detailed DFU severity measures (infection severity, ischemia grade, ulcer size, osteomyelitis, and systemic illness) were also unavailable. Wagner grade alone cannot capture these dimensions.

Second, Mg was a single total-serum measurement; acute infection, hydration, renal function, nutrition, and treatment timing could influence it, and serum Mg may not reflect chronic or intracellular status. The exact pre-treatment sampling interval and local reference interval require confirmation. Third, 373 screened patients lacking Mg were excluded, but their baseline records were unavailable for comparison, creating possible selection bias. Fourth, MissForest produced one imputed dataset rather than formal multiple imputation, although the complete-case analysis was consistent. Fifth, the endpoint file did not separate nonfatal MI from stroke or retain detailed diagnostic/adjudication fields or a loss-to-follow-up indicator. Sixth, nonproportional hazards and small late risk sets complicate a single summary HR. Finally, spline and change-point analyses were data-driven, and the matched cutoff used the outcome; neither was internally or externally validated. Generalizability beyond this predominantly Chinese single-center cohort is uncertain.

## 6 Conclusion

Among patients with active DFU, baseline total serum Mg was independently associated with MACE risk, and the association showed an exploratory nonlinear pattern. The observed change points near 0.83 and 0.99 mmol/L are uncertain, data-derived values—not therapeutic boundaries or evidence for supplementation. External prospective validation and intervention studies are required before serum Mg can be used as a treatment target.

## Data Availability

The raw data supporting the conclusions of this article will be made available by the authors, without undue reservation.

## Supplementary material

Supplementary Figure 1 Love plot of absolute standardized mean differences before and after propensity-score matching. The dashed line marks |SMD|=0.10. All post-match absolute SMDs were <0.10.
