# Supplementary material for: Serum magnesium and major adverse cardiovascular events in patients with diabetic foot ulcers: a retrospective cohort study
Source: Front Endocrinol (Lausanne). 2026 Sep 11;17:1938560. doi: 10.3389/fendo.2026.1938560 (PMC13612363; doi:10.3389/fendo.2026.1938560)
Supplement: Supplementary file 1 [file Table1.docx]

**Supplementary Table S1. Missingness before MissForest single imputation**

| Variable | Missing n | Missing % |
| --- | --- | --- |
| Diabetic nephropathy | 297 | 25.3% |
| HbA1c | 220 | 18.7% |
| BMI | 205 | 17.4% |
| CRP | 70 | 6.0% |
| SOD | 23 | 2.0% |
| Total cholesterol | 8 | 0.7% |
| LDL-C | 4 | 0.3% |
| Triglycerides | 3 | 0.3% |
| Serum creatinine | 3 | 0.3% |
| eGFR | 3 | 0.3% |
| TyG index | 3 | 0.3% |
| HDL-C | 3 | 0.3% |
| Renal Group | 3 | 0.3% |
| Albumin | 2 | 0.2% |

Serum Mg, MACE status, and follow-up time had no missing values in the analyzed cohort and were not imputed. MissForest created one completed dataset; no multiple-imputation pooling was performed.

**Supplementary Table S2. Covariate balance before and after propensity-score matching**

| Characteristic | Before: low | Before: high | SMD | After: low | After: high | SMD |
| --- | --- | --- | --- | --- | --- | --- |
| Age, years | 60.55±11.40 | 62.52±11.63 | 0.171 | 60.90±11.29 | 61.01±11.38 | 0.009 |
| Male sex | 224 (67.07) | 626 (74.35) | 0.160 | 222 (68.52) | 229 (70.68) | 0.047 |
| BMI, kg/m² | 24.52±3.53 | 24.58±3.22 | 0.017 | 24.46±3.52 | 24.63±3.00 | 0.049 |
| Smoking | 135 (40.42) | 353 (41.92) | 0.031 | 134 (41.36) | 138 (42.59) | 0.025 |
| Duration of diabetes, years | 15.01±8.23 | 15.12±9.03 | 0.013 | 15.02±8.17 | 14.98±8.75 | -0.004 |
| Ulcer duration, months | 94.33±175.99 | 110.54±222.83 | 0.081 | 92.77±169.42 | 78.75±122.16 | -0.095 |
| DPN | 295 (88.32) | 745 (88.48) | 0.005 | 285 (87.96) | 286 (88.27) | 0.010 |
| PAD | 225 (67.37) | 613 (72.80) | 0.119 | 220 (67.90) | 213 (65.74) | -0.046 |
| DR | 179 (53.59) | 433 (51.43) | -0.043 | 172 (53.09) | 167 (51.54) | -0.031 |
| DN | 209 (62.57) | 467 (55.46) | -0.145 | 203 (62.65) | 204 (62.96) | 0.006 |
| Hypertension | 244 (73.05) | 574 (68.17) | -0.107 | 235 (72.53) | 245 (75.62) | 0.070 |
| Coronary heart disease | 95 (28.44) | 231 (27.43) | -0.022 | 95 (29.32) | 97 (29.94) | 0.013 |
| Previous stroke | 59 (17.66) | 188 (22.33) | 0.117 | 59 (18.21) | 65 (20.06) | 0.047 |
| Hyperlipidemia | 250 (74.85) | 580 (68.88) | -0.133 | 243 (75.00) | 249 (76.85) | 0.043 |
| HbA1c, % | 9.49±2.08 | 8.82±1.78 | -0.344 | 9.41±2.05 | 9.22±1.72 | -0.100 |
| FPG, mmol/L | 8.95±3.57 | 8.54±3.58 | -0.113 | 8.87±3.51 | 8.78±3.46 | -0.026 |
| Serum creatinine, µmol/L | 79.40±58.10 | 111.45±153.58 | 0.276 | 79.87±58.65 | 77.16±40.96 | -0.053 |
| eGFR, mL/min/1.73 m² | 90.31±26.25 | 83.40±30.38 | -0.244 | 90.02±26.15 | 89.92±24.68 | -0.004 |
| Serum uric acid, µmol/L | 299.32±101.55 | 309.95±105.85 | 0.102 | 299.25±102.53 | 301.13±104.09 | 0.018 |
| Total cholesterol, mmol/L | 3.77±1.09 | 3.93±1.03 | 0.152 | 3.78±1.10 | 3.75±0.95 | -0.029 |
| Triglycerides, mmol/L | 1.44±0.74 | 1.52±1.08 | 0.090 | 1.43±0.74 | 1.40±0.64 | -0.056 |
| HDL-C, mmol/L | 0.89±0.28 | 0.95±0.28 | 0.209 | 0.90±0.28 | 0.90±0.26 | -0.007 |
| LDL-C, mmol/L | 2.19±0.81 | 2.29±0.80 | 0.125 | 2.20±0.82 | 2.21±0.75 | 0.012 |
| Albumin, g/L | 35.30±5.57 | 37.43±5.04 | 0.400 | 35.45±5.54 | 35.58±5.23 | 0.024 |
| CRP, mg/L | 41.97±51.26 | 32.87±49.25 | -0.181 | 41.68±51.63 | 41.21±53.34 | -0.009 |
| SOD, U/mL | 109.50±28.93 | 113.76±27.23 | 0.152 | 109.61±29.05 | 108.86±26.61 | -0.027 |
| TyG index | 9.05±0.64 | 9.03±0.67 | -0.040 | 9.04±0.64 | 9.02±0.59 | -0.027 |
| Wagner grade 1 | 17 (5.09) | 31 (3.68) | -0.069 | 17 (5.25) | 14 (4.32) | -0.043 |
| Wagner grade 2 | 40 (11.98) | 131 (15.56) | 0.104 | 40 (12.35) | 39 (12.04) | -0.009 |
| Wagner grade 3 | 86 (25.75) | 238 (28.27) | 0.057 | 84 (25.93) | 85 (26.23) | 0.007 |
| Wagner grade 4-5 | 191 (57.19) | 442 (52.49) | -0.094 | 183 (56.48) | 186 (57.41) | 0.019 |

Low Mg: ≤0.80 mmol/L; high Mg: >0.80 mmol/L. Post-match: 324 pairs.

Continuous variables are mean±SD; binary variables are n (%). Absolute SMD <0.10 was the balance target. Maximum post-match |SMD|=0.100 after rounding (unrounded maximum 0.0999).

**Supplementary Table S3. Sensitivity analyses for MACE per 0.1 mmol/L higher serum magnesium**

| Analysis | Exposure contrast | n | Events | HR (95% CI) | p |
| --- | --- | --- | --- | --- | --- |
| Complete-case extended Model 3 | Per 0.1 mmol/L | 598 | 141 | 0.85 (0.75–0.97) | 0.015 |
| Exclude eGFR <30 | Per 0.1 mmol/L | 1,101 | 272 | 0.80 (0.72–0.89) | <0.001 |
| eGFR <30 stratum | Per 0.1 mmol/L | 75 | 32 | 0.87 (0.65–1.17) | 0.355 |
| eGFR 30-59 stratum | Per 0.1 mmol/L | 140 | 46 | 0.85 (0.64–1.14) | 0.290 |
| eGFR >=60 stratum | Per 0.1 mmol/L | 961 | 226 | 0.81 (0.72–0.91) | <0.001 |
| No previous CHD/stroke | Per 0.1 mmol/L | 710 | 152 | 0.82 (0.71–0.95) | 0.008 |
| Previous CHD/stroke | Per 0.1 mmol/L | 466 | 152 | 0.90 (0.81–1.01) | 0.075 |
| Fine-Gray subdistribution hazard extended Model 3 | Per 0.1 mmol/L | 1,176 | 304 | 0.91 (0.83–0.99) | 0.034 |
| Time-specific Cox: 0-2 years | Per 0.1 mmol/L | 1,176 | 82 | 0.93 (0.80–1.08) | 0.335 |
| Time-specific Cox: >2 years | Per 0.1 mmol/L | 869 | 222 | 0.84 (0.75–0.93) | 0.001 |
| PSM continuous Mg; Cox stratified by matched pair | Per 0.1 mmol/L | 648 | 167 | 0.85 (0.71–1.01) | 0.070 |
| PSM high vs low Mg; Cox stratified by matched pair | >0.80 vs ≤0.80 mmol/L | 648 | 167 | 0.60 (0.39–0.92) | 0.020 |

Fine–Gray estimates are subdistribution HRs with noncardiovascular death as the competing event. PSM Cox models were stratified by matched pair. Renal-stratum and previous-CVD models used the Model 3 covariate set where estimable.

**Supplementary Table S4. Proportional-hazards tests for the extended Model 3**

| Term | Chi-square | df | p |
| --- | --- | --- | --- |
| Serum Mg | 5.09 | 1 | 0.024 |
| Age | 0.28 | 1 | 0.599 |
| BMI | 0.48 | 1 | 0.488 |
| Duration of diabetes | 0.17 | 1 | 0.679 |
| Ulcer duration | 0.13 | 1 | 0.720 |
| Female sex | 0.77 | 1 | 0.380 |
| Smoking | 0.03 | 1 | 0.868 |
| DPN | 14.95 | 1 | <0.001 |
| PAD | 0.56 | 1 | 0.456 |
| DR | 0.26 | 1 | 0.612 |
| DN | 1.73 | 1 | 0.188 |
| Hypertension | 0.67 | 1 | 0.411 |
| CHD | 0.18 | 1 | 0.674 |
| Previous stroke | 0.29 | 1 | 0.590 |
| Hyperlipidemia | 0.04 | 1 | 0.839 |
| Wagner grade | 2.99 | 3 | 0.393 |
| HbA1c | 0.25 | 1 | 0.617 |
| eGFR | 7.96 | 1 | 0.005 |
| Albumin | 2.91 | 1 | 0.088 |
| log(CRP+1) | 5.74 | 1 | 0.017 |
| HDL-C | 0.82 | 1 | 0.365 |
| LDL-C | 1.44 | 1 | 0.231 |
| Global | 45.32 | 24 | 0.005 |

Scaled Schoenfeld-residual score tests. The primary Cox HR is interpreted as an average association because serum Mg and the global model showed evidence of nonproportionality.

**Supplementary Table S5. Serum magnesium upper-tail distribution**

| Serum Mg, mmol/L | Participants | MACE | MACE % |
| --- | --- | --- | --- |
| ≥1.00 | 383 | 105 | 27.4% |
| ≥1.01 | 186 | 60 | 32.3% |
| ≥1.05 | 174 | 57 | 32.8% |
| ≥1.10 | 170 | 56 | 32.9% |
| ≥1.20 | 39 | 19 | 48.7% |
| ≥1.30 | 5 | 4 | 80.0% |

These are descriptive counts, not causal contrasts. Sparse observations at ≥1.20 and ≥1.30 mmol/L limit precision in the upper tail.

**Supplementary Table S6. Exploratory two-change-point Cox model**

| Segment | n | MACE | HR per 0.1 mmol/L (95% CI) | p |
| --- | --- | --- | --- | --- |
| <0.83 mmol/L | 360 | 95 | 1.17 (0.90–1.51) | 0.245 |
| 0.83 to <0.99 mmol/L | 426 | 104 | 0.51 (0.40–0.66) | <0.001 |
| ≥0.99 mmol/L | 390 | 105 | 1.28 (1.03–1.60) | 0.024 |

The selected change points were 0.83 and 0.99 mmol/L; approximate 95% profile-likelihood regions were 0.75–0.85 and 0.95–1.10 mmol/L. The descriptive likelihood-ratio comparison with a linear Mg model gave p<0.001. Because thresholds were selected in the same dataset and were not internally or externally validated, they should not be interpreted as precise clinical boundaries.
